# Photopolymerization-Based Synthesis of Uniform Magnetic Hydrogels and Colorimetric Glucose Detection

**DOI:** 10.3390/ma13194401

**Published:** 2020-10-02

**Authors:** Seok Joon Mun, Donghyun Ko, Hyeon Ung Kim, Yujin Han, Yoon Ho Roh, Bong-Geun Kim, Hyon Bin Na, Ki Wan Bong

**Affiliations:** 1 Department of Chemical and Biological Engineering, Korea University, 145 Anam-ro, Seongbuk-gu, Seoul 02841, Korea; msj95@naver.com (S.J.M.); rlagus2009@gmail.com (H.U.K.); yoonho90@korea.ac.kr (Y.H.R.); 2 Department of Chemical Engineering, Myongji University, Yongin, Gyeonggi-do 17058, Korea; rhehd1992@naver.com (D.K.); padug2@naver.com (Y.H.); persson@naver.com (B.-G.K.)

**Keywords:** magnetic hydrogels, photoinitiator absorbance, peroxidase mimics, colorimetric detection

## Abstract

Magnetic hydrogels have been commonly used in biomedical applications. As magnetite nanoparticles (MNPs) exhibit peroxidase enzyme-like activity, magnetic hydrogels have been actively used as signal transducers for biomedical assays. Droplet microfluidics, which uses photoinitiated polymerization, is a preferred method for the synthesis of magnetic hydrogels. However, light absorption by MNPs makes it difficult to obtain fully polymerized and homogeneous magnetic hydrogels through photoinitiated polymerization. Several methods have been reported to address this issue, but few studies have focused on investigating the light absorption properties of photoinitiators. In this study, we developed a simple method for the synthesis of poly(ethylene glycol) (PEG)-based uniform magnetic hydrogels that exploits the high ultraviolet absorption of a photoinitiator. Additionally, we investigated this effect on shape deformation and structural uniformity of the synthesized magnetic hydrogels. Two different photoinitiators, Darocur 1173 and lithium phenyl (2,4,6–trimethylbenzoyl) phosphinate (LAP), with significantly different UV absorption properties were evaluated based on the synthesis of magnetic hydrogels. The magnetic characteristics of the PEG-stabilized MNPs in hydrogels were investigated with a vibrating sample magnetometer. Finally, the colorimetric detection of hydrogen peroxide and glucose was conducted based on the enzyme-like property of MNPs and repeated several times to observe the catalytic activity of the magnetic hydrogels.

## 1. Introduction

Hydrogels embedded with magnetite nanoparticles (MNPs) have gained much attention because of the following: (1) tunable mechanical and chemical properties of polymer gel particles, and (2) diverse functions of MNPs, such as remote controllability, rapid response, heat generation, and catalytic activities. Owing to these features, magnetic hydrogels have been extensively used in biomedical applications, including drug delivery systems [1,2], tissue engineering [3], hyperthermia [4,5,6], immunoassays [7], and biosensors [8,9]. In particular, magnetic hydrogels have been investigated as signal transducers for biomedical assays, as MNPs exhibit peroxidase enzyme-like activity with high chemical and biological stability [10]. MNPs can catalyze the oxidation of substrates with hydrogen peroxide, which can be applied in colorimetric assays [11,12] and for the electrochemical detection of pathogens [13]. In addition to the high catalytic stability and low manufacturing cost of MNPs, the inherent magnetic properties and ease of modification are expected to provide additional functionalities and enhance detection efficiency [14]. Biomedical assays are usually conducted in physiological media; thus, nanoparticles with enzyme-like activity and nanozymes are also dispersed in solution. Because catalytic reactions occur on the surface of nanoparticles, the dispersity of nanoparticles in aqueous media is an important factor for maximizing catalytic activities [15]. As one strategy to maintain stable nanoparticle dispersion without aggregation or sedimentation in physiological environments, incorporation into hydrogels that provide a crosslinked and flexible matrix for MNPs has been demonstrated [8,16].

A variety of methods such as flow lithography [17], molding lithography [18], as well as suspension and emulsion methods [19] have been developed to synthesize magnetic hydrogels for decades. Among them, macro-, mini-, micro-emulsion, and suspension methods have been commonly used, owing to their simplicity in generating emulsion droplets by agitating two immiscible phases mechanically and/or manually. These bulk synthesis methods, however, have intrinsic size limitations on the generation of monodisperse emulsions due to the non-uniform distribution of shear stresses applied across the system [19,20]. Furthermore, the homogeneous distribution of inorganic materials within the polymer matrix is often difficult to obtain, especially for MNPs [21,22], which causes the magnetic hydrogels to exhibit inconsistent behaviors. Other techniques, such as viscoelastic shear [23,24], membrane extrusion [25,26], and microthread generation [27], have been used to reduce the size distribution and surface treatment steps to achieve a more homogeneous distribution of MNPs, but further improvements in hydrogel particle monodispersity and the uniform MNP distribution are needed for their effective use in biomedical assays.

Droplet microfluidics has been widely used to synthesize gel microparticles due to the generation of monodisperse emulsion droplets in a simple yet reproducible format [28,29]. In droplet microfluidics, two immiscible fluids (a dispersed and a continuous phase) interact to induce droplet formation. At the point of intersection in the microfluidic channel, the dispersed phase encounters the continuous phase and separates into discrete emulsions by shearing force. As liquid emulsions have a property to minimize their interfacial free energy, spherical droplet emulsions are usually formed in the channel. Droplet emulsions can also exhibit plug or disc shapes based on droplet size and channel geometry in the microfluidic device [30,31]. The size of the droplets can be simply manipulated by modulating the relative flow rates of the two fluids, with a minimum droplet diameter of several hundred nanometers [32]. When the flow rates of the two phases are fixed, the morphology of sheared droplets remains nearly constant, which enables fabrication of monodisperse emulsion droplets.

To cure the droplets into a gel matrix, ultraviolet (UV) radiation and thermal energy are commonly used with either UV or thermally curable polymers. In microfluidics, UV-induced polymerization is generally preferred over thermal polymerization because of its faster processing times, which enables homogeneous distribution of inorganic materials in the polymer as well as the uniform structural formation of hydrogels without the requirement of high-temperature processing or organic solvents. However, MNPs exhibit strong UV absorption due to their opacity, making it difficult for photoinitiators to absorb sufficient energy for photolysis into radicals. As a result, radical polymerization is difficult to initiate in emulsion droplets, and the resulting magnetic hydrogels frequently exhibit shape deformation, partial polymerization, and inhomogeneous distribution of MNPs. One potential method to address this issue involved the insertion of aluminum foil as a UV reflector into a microfluidic channel, with the increased net energy flux and multidirectional irradiation of UV rays enabling full polymerization of magnetic hydrogels [33]. However, this method requires both the fabrication of an additional microfluidic device and the precise (and repeatable) placement of aluminum foil above the channel geometry without consideration to other potential factors contributing to the synthesis of magnetic hydrogels, such as photoinitiator absorbance.

We introduce a simple method for the synthesis of uniform magnetic hydrogels by exploiting the high UV absorption of a photoinitiator. Furthermore, we investigated the effect of absorbance on shape deformation and structural uniformity of the magnetic hydrogels. Two different photoinitiators exhibiting significant differences in absorbance were evaluated based on the synthesis of the magnetic hydrogels. The magnetic behavior of the synthesized magnetic hydrogels was also investigated under an applied magnetic field with a vibrating sample magnetometer. Finally, the colorimetric detection of hydrogen peroxide and glucose was conducted based on the enzyme-like property of MNPs and repeated several times to observe the catalytic activity of the magnetic hydrogels.

## 2. Materials and Methods

### 2.1. Microfluidic Device

The top layer of the polydimethylsiloxane (PDMS) block was patterned with a T-junction droplet microfluidic channel with a height of 100 μm. This top layer was fabricated by pouring PDMS (SYLGARD 184, Corning, Midland, MI, USA), mixed in a 10:1 (base:curing agent) ratio by weight, over an SU-8 master and curing for 3 h in an oven at 70 °C. After peeling off the cured PDMS replica from the mold, two inlet holes and one outlet hole were perforated using 1 mm punches supplied by Miltex (Princeton, NJ, USA) and sonicated in ethanol for 10 min for cleaning. The PDMS microchannel was sealed by bonding the top layer of the PDMS block with PDMS-coated slide glass utilizing a vacuum plasma (CUTE, Femtoscience, Hwaseong-si, Gyeonggi-do, Korea). The PDMS microfluidic device was then baked at 373 °C for 3 h to return the surface of the channel to its hydrophobic state.

### 2.2. Materials

Water-dispersible magnetite nanoparticles (MNPs) were prepared through surface modification of oleic acid-stabilized magnetite nanoparticles (OA–Fe_3_O_4_ NPs) with poly(ethylene glycol) (PEG)-based hydrophilic ligands (Figure S1) [34,35]. OA–Fe_3_O_4_ NPs were synthesized by a thermal decomposition of iron-oleate precursor in 1–octadecene. MNPs were prepared by further modification of OA–Fe_3_O_4_ NPs with phosphine oxide-functionalized PEG (PO–PEG). In a typical ligand exchange reaction, 50 mg of OA–Fe_3_O_4_ NPs was dispersed in a tetrahydrofuran (THF) solution containing 280 mg of PO–PEG ligand. After removing THF under vacuum, the NP-ligand mixture was incubated at 80 °C under vacuum for 4 h. The resulting paste was dispersed in 5 mL of deionized (DI) water and then filtered through a 0.45 μm syringe filter. The MNPs were further purified with excess DI water using a centrifugal filter (molecular weight cutoff: 50 kDa, Merck Millipore, Burlington, MA, USA).

Two photoinitiators, lithium phenyl (2,4,6–trimethylbenzoyl) phosphinate (LAP, TCI, Tokyo, Japan) and 2–hydroxy–2–methylpropiophenone (Darocur 1173, Sigma-Aldrich, St. Louis, MO, USA) were used to manufacture the dispersed phases. The MNP solution, poly(ethylene glycol) (700) diacrylate (PEG-DA 700, Sigma-Aldrich, St. Louis, MO, USA), and DI water were mixed in a volume ratio of 4:4:2 with 0.5% (w/v) LAP for the first phase. For the second phase, the MNP solution, poly(ethylene glycol) (700) diacrylate, and DI water were mixed in a volume ratio of 8:8:3 with 5% (v/v) Darocur 1173. Although Darocur 1173 has low water solubility, it was able to be well-solubilized in dispersed phase without phase separation due to the amphiphilicity of PEG-DA 700. A mixture of 92% mineral oil (Sigma-Aldrich, St. Louis, MO, USA) (v/v) and 8% ABIL^®^ EM 90 (Evonik Industries AG, Essen, Germany) (v/v) was used as a continuous phase. For the structural uniformity observation of magnetic hydrogels, 10 mg/mL of 2% (v/v) methacryloxyethyl thiocarbamoyl rhodamine B (Polysciences, Inc., Warrington, PA, USA) was added to each manufactured dispersed phase.

2,2–Azino–bis (3–ethylbenzothiazoline–6–sulfonic acid) diammonium salt (ABTS) was purchased from Sigma-Aldrich (St. Louis, MO, USA), and 35% (w/w) of hydrogen peroxide solution was purchased from Alfa Aesar (Ward Hill, MA, USA).

### 2.3. Characterization

The morphology and hydrodynamic size of the nanoparticles were characterized through transmission electron microscopy (JEM-2011, JEOL, Tokyo, Japan) and dynamic light scattering (Zetasizer Nano ZS, Malvern Instruments, Malvern, Worcestershire, UK), respectively. The concentration of Fe in the dispersion was analyzed through atomic absorption spectroscopy (AA-7000, Shimadzu, Tokyo, Japan).

The absorbance of all the materials, deionized water, MNP solution, monomers, and photoinitiators, which comprise the dispersed phase, were characterized using the UV absorbance mode of a SpectraMax id5 multimode microplate reader (Molecular Devices, San Jose, CA, USA). The entire process was performed on a clear 96-well plate (Corning, Costar, Corning, NY, USA) at 25 °C. Measurements were taken at intervals of 1 nm from UV wavelengths of 300 and 500 nm.

Spectrophotometric measurements in assays were taken using a microplate reader (Spark, Tecan, Meilen, Zurich, Switzerland) and a UV–VIS spectrophotometer (S-3100, Scinco, Seoul, Korea).

### 2.4. Generation of Emulsion Droplets

Spherical magnetic hydrogels with a hydrodynamic diameter of 30 μm were generated from two immiscible flows driven by compressed air and polymerized via UV-initiated photopolymerization in a PDMS microfluidic channel. A UV lamp (Illuminator HXP 120V, Zeiss, Oberkochen, Land Baden-Württemberg, Germany) was used as a light source to illuminate light with a broad wavelength spectrum (280 to 880 nm). The desired UV wavelength band (365 to 390 nm) was selected and exposed to target areas by filtering the light through a UV filter set (Chroma 19000, Chroma, Bellows Falls, VT, USA) and a 20× microscope objective (Zeiss, Oberkochen, Land Baden-Württemberg, Germany). The exposed area was regulated by an aperture in an inverted microscope (Axio Observer 3, Zeiss, Oberkochen, Germany). The flow rates of the two phases were properly manipulated using air pressure regulators (type 70, Marsh Bellofram, Newell, WV, USA), thereby allowing the formed droplets to absorb UV light for more than 4.0 s. The entire synthesis process was observed using a Canon EOS 6D camera (Canon, Tokyo, Japan).

### 2.5. Vibrating Sample Magnetometer Measurement

The magnetizations of freeze-dried MNPs and magnetic hydrogels were measured using a vibrating sample magnetometer (VSM) equipped in a physical property measurement system (Quantum Design Inc., San Diego, CA, USA). In the VSM, the samples are first magnetized in a uniform magnetic field and then vibrated sinusoidally. This generates changes in the magnetic flux near the coil and induces a voltage, which is ultimately measured and indicated in digital figures. By applying a full cycle of the magnetic fields (−1.0 to +1.0 T) to the samples, their magnetization values were obtained.

### 2.6. Colorimetric Detection of H_2_O_2_ and Glucose

The detection of H_2_O_2_ was conducted to investigate the peroxidase-like activity of the magnetic hydrogels. The catalytic oxidation of ABTS in the presence of H_2_O_2_ with various concentrations was performed in a 96-well plate. A solution containing 140 μL of sodium acetate buffer (0.2 M, pH of 4.0), 20 μL of magnetic hydrogels (0.2 Fe mg/mL), 20 μL of H_2_O_2_ solution, and 20 μL of ABTS (60 mM) was added into the wells, and the plate was incubated at room temperature (RT) for 20 min. After the reaction, the magnetic hydrogels were separated from the mixtures by the application of an external magnetic field for 10 s. Subsequently, 100 μL of the supernatant was transferred to a new well, and the absorption spectrum was measured.

The reusability of the magnetic hydrogels was investigated by the catalytic oxidation of ABTS in the presence of H_2_O_2_. A solution containing 420 μL of sodium acetate buffer (0.2 M, pH of 4.0), 60 μL of magnetic hydrogels (0.2 Fe mg/mL), 60 μL of H_2_O_2_ solution (5 mM), and 20 μL of ABTS (60 mM) was added into a microtube and incubated at RT for 10 min. After the reaction, the magnetic hydrogels were separated from the mixtures through the application of an external magnetic field for 15 s. Subsequently, 400 μL of the supernatant was collected, and the absorbance at 417 nm was measured. After washing with DI water by vortexing for 2 min, the magnetic hydrogels were reused for the next catalytic oxidation under the same conditions.

The detection of glucose was performed in a 96-well plate as follows. A total of 20 μL of glucose oxidase (GOx, 10 mg/mL) and 100 μL of glucose solution with a pre-determined concentration were first incubated in phosphate-buffered saline (10 mM, pH of 7.0) at RT for 20 min. Then, 20 μL of solution was transferred into another well containing 140 μL of sodium acetate buffer (0.2 M, pH of 4.0), 20 μL of magnetic hydrogels (0.2 Fe mg/mL), and 20 μL of ABTS (60 mM). After shaking at RT for 40 min, the magnetic hydrogels were separated from the mixture through the application of an external magnetic field for 10 s. Then, 100 μL of the supernatant was transferred to a new well, and the absorption spectrum was measured. A photographic image was taken using a smartphone (Galaxy S9, Samsung Electronics, Suwon-si, Korea) and converted into a grayscale image using the software package ImageJ (version of 1.52a, National Institutes of Health, Bethesda, MD, USA). After inversion, the intensity of the wells was measured.

## 3. Results and Discussion

### 3.1. Synthesis of Magnetic Hydrogels

Photoinitiator absorbance, precursor composition, UV intensity, and UV exposure time are important factors to consider in the polymerization of magnetic hydrogels [36,37,38]. Among these factors, photoinitiator absorbance is the only one that is directly affected by UV absorption of MNPs, where the initiation of radical polymerization is significantly reduced. This makes it difficult for magnetic hydrogels to be fully polymerized and frequently results in other problems such as shape deformation and nonuniform structure formation. Thus, we investigated how UV absorbance influences the morphology and structural uniformity of the synthesized magnetic hydrogels based on using two photoinitators with large differences in their UV absorption properties.

**Figure 1:**
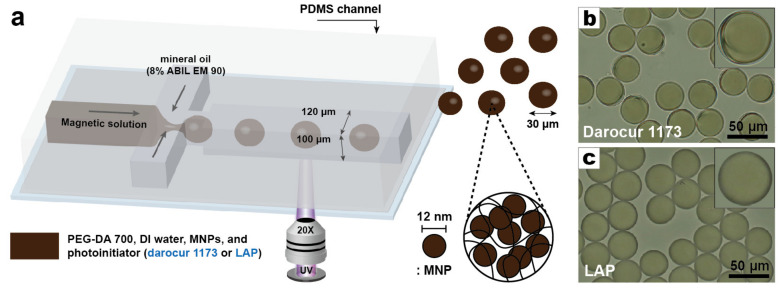
Droplet microfluidics for synthesis of magnetic hydrogels and effect of photoinitiator absorbance on morphology of magnetic hydrogel. (**a**) Droplet generation and particle synthesis in a droplet microfluidic device. Magnetic hydrogels synthesized using: (**b**) Darocur 1173, or (**c**) lithium phenyl (2,4,6–trimethylbenzoyl) phosphinate (LAP) dispersed in DI water.

Darocur 1173, which is a commonly used photoinitiator and known to have an absorption wavelength band (240 to 340 nm) poorly matched with the wavelength band of irradiated light (365 to 390 nm), was first exploited for the synthesis of magnetic hydrogels (Figure 1a). The UV exposure time was set to ~5.0 s by controlling the flow rates of the continuous phase and the UV exposed area independently. With a UV intensity of 61.9 mW·cm^2^, emulsion droplets (30-μm hydrodynamic diameter) were continually generated by adjusting and then setting the flow rates of both dispersed and continuous fluids. After several rinse steps, the magnetic hydrogels were observed through a 20× microscope objective dispersed in water (Figure 1b). Shape deformation from the initial spherical geometry was confirmed in most of the magnetic hydrogels. In addition, partial polymerization could be observed in certain areas of the magnetic hydrogels, particularly where deformation had occurred. A second photoinitiator, LAP, was used to fabricate magnetic hydrogels under the same synthetic conditions (Figure 1c). LAP exhibits high absorbance near 370 nm, which is better matched with the irradiated light than Darocur 1173. Magnetic hydrogels synthesized with LAP did not exhibit any shape deformation or partial polymerization. The size distribution of the magnetic hydrogels was also very narrow (CV ~ 0.3%). For the quantitative analysis of shape deformation, the circularity of the magnetic hydrogels in Figure 1a,b each was measured via the ImageJ software (version of 1.52a, National Institutes of Health, Bethesda, MD, USA) (Figure S2). In consequence, magnetic hydrogels in Figure 1b showed 1.2 times enhanced averaged circularity than those in Figure 1a, which means LAP exhibits 1.2 times better performance in retaining the morphology than Darocur 1173. Leaking of Darocur 1173 to continuous phase owing to its low water solubility may be thought to increase the shape deformations and lack of structural uniformity. However, because the difference in water solubility is small between two photoinitiators, 0.08 M for Darocur 1173 and 0.1 M for LAP, and the molar concentration of Darocur 1173 in the dispersed phase is higher than that of LAP, leaking of Darocur 1173 is estimated to have much less effect on shape deformation than LAP. This difference in magnetic hydrogel synthesis with these two photoinitiators suggests that photoinitiator absorbance can play a significant role in the degree of polymerization and hydrogel morphology. This is also supported by the following equation [39].

(1) $$R_{p}=\left(\frac{k_{p}}{k_{t}^{0.5}}\right){\left(\emptyset_{diss}\emptyset_{RM}I_{abs}\right)}^{0.5}\left[M\right],$$

where *R_p_* is the rate of polymerization, *k_p_* is the propagation rate constant, *k_t_* is the termination rate constant, $\emptyset_{diss}$ is the dissociation quantum yield, $\emptyset_{RM}$ is the yield in the first monomer radical, *I_abs_* is the absorbance of photoinitiator, and [*M*] is the monomer concentration. As the monomer was not changed and its concentration in the precursor was sufficiently high (40% (v/v) of the total volume), the values of the propagation rate constant, termination rate constant, yield in the first monomer radical, and monomer concentration are constant for both photoinitiators [40]. In addition, as Darocur 1173 and LAP have almost the same dissociation quantum yield, which is 0.38 and 0.35, respectively, the polymerization rate is only proportional to the square root of the absorbance. From this correlation between polymerization rate and absorbance of the photoinitiator, we can infer that increased absorption of LAP enabled full polymerization of magnetic hydrogels without any shape deformation. The maximum concentration of the MNPs that can be encapsulated in hydrogels was confirmed to be 4 mg Fe/mL. Above this concentration, the shape deformation increased and the circularity decreased in magnetic hydrogels, regardless of the photoinitiator used (Figure S3).

### 3.2. Absorbance Characterization

**Figure 2:**
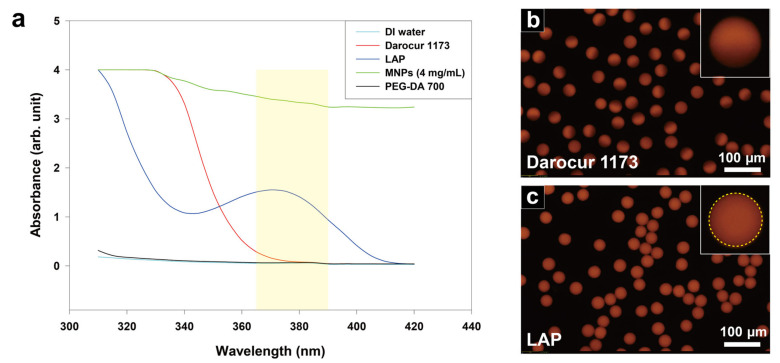
Effect of photoinitiator absorbance on magnetic hydrogel polymerization. (**a**) Absorbance spectra of components used in dispersed phase fluid. The wavelength band of irradiated light is shaded in yellow. (**b**) Fluorescence image of magnetic hydrogels polymerized with Darocur 1173. (**c**) Fluorescence image of magnetic hydrogels polymerized with LAP.

To measure the absorbance of the materials that compose the magnetic hydrogels, especially the photoinitiators, UV–visible near infrared spectroscopy was used (Figure 2a). Each material was dissolved in ethanol as a basic solvent while maintaining its composition as mixed in the precursor. The measured values were subtracted from the reference value of ethanol to compensate for the effect of the solvent during the measurement. MNPs exhibit the highest absorbance over the entire wavelength range, whereas the monomer (PEG-DA 700) and water exhibit little to no absorbance. Darocur 1173 exhibits high absorbance only up to 350 nm, while LAP exhibits much higher absorbance in the wavelength band of irradiated light (with a spectral peak at 370 nm). The absorption spectra of these photoinitiators are similar to those reported in previous studies [41,42]. The average absorbance of both photoinitiators within the wavelength band of irradiated light was calculated based on dividing the area under the absorbance curve by the width of the wavelength band (25 nm). LAP exhibited an absorbance 12 times higher than that of Darocur 1173, which indicates that 12 times more photons were absorbed by LAP under exposure to the same intensity of light. Based on Equation (1), the polymerization rate of magnetic hydrogels using LAP (relative to Darocur 1173) is 3.5 times faster. Assessment of the synthesized magnetic hydrogels indicate that the improved polymerization rate was sufficient to enable full polymerization of the magnetic hydrogels.

### 3.3. Uniformity of the Magnetic Hydrogel

To further assess structural uniformity of magnetic hydrogels, a functionalized fluorescent dye was added to the original precursor to synthesize magnetic hydrogels. The fluorescent dye has an acrylate group and a fluorescent body at each end of the chemical structure. Because monomers (PEG-DA700) have a functional group of acrylates and fluorescent dyes have a functional group of methacryloxy group, they are covalently bonded during polymerization. As the degree of polymerization increases, more fluorescent dye reacts with the monomer, and a stronger fluorescence signal is generated. The structural uniformity of magnetic hydrogels can be observed by measuring the fluorescence intensity along a given microparticle dimension. A sharp fluorescence intensity gradient was observed in most of the magnetic hydrogels polymerized with Darocur 1173 (Figure 2b). This result indicates significant variability in the degree of polymerization for a given droplet emulsion when using this photoinitiator. Furthermore, it is attributed to a large reduction in the number of photons by MNPs which occurs as the UV light passes through the magnetic hydrogels. This results in a smaller amount of available energy along the path vertically from the bottom surface to the top surface of the droplet emulsion. A nearly uniform fluorescence intensity profile was observed in most of the magnetic hydrogels polymerized with LAP (Figure 2c). The minimal difference in fluorescence intensity across the particle dimension indicates the nearly uniform polymerization of droplet emulsions into magnetic hydrogels. We measured the fluorescence intensity of individual magnetic hydrogels in Figure 2b,c each via the ImageJ software and generated a dot plot to observe the distribution of the signals (Figure S4). Consequently, the magnetic hydrogels in Figure 2c showed 1.2 times higher fluorescence intensity on average and 0.9 times less standard deviation than those in Figure 2b. It verifies the better performance of LAP than Darocur 1173 in terms of degree of polymerization and uniformity of the magnetic hydrogels.

### 3.4. Magnetic Characterization of Magnetic Hydrogels

The magnetic properties of both MNPs and magnetic hydrogels were characterized using VSM measurements (Figure S5). For a MNP concentration of 4 mg Fe/mL, magnetic hydrogels were synthesized with LAP, and VSM measurements were conducted at RT under an applied magnetic field between −1.0 and +1.0 T. Magnetic hydrogels showed superparamagnetic behavior without any hysteresis or coercivity observed. This result suggests that neither agglomeration nor sedimentation has occurred in the MNPs or magnetic hydrogels, as superparamagnetism only appears in sufficiently small MNPs. The saturation magnetization value was calculated using the following equation [33].

(2) $$M=M_{S0}\left(1-\frac{6}{\pi }\frac{k_{b}T}{M_{s}d^{3}B}\right)$$

where *M* is the magnetization value measured by VSM, $M_{S0}$ is the saturation magnetization value, and *B* is the applied magnetic field. The saturation magnetization value was obtained by plotting and linearly fitting a line of *M* versus 1/*B* at a large magnetic field from 0.2 to 0.5 T. The computed saturation magnetization values of the MNPs and magnetic hydrogel were 26.2125 A·m^2^ kg^−1^ and 0.5865 A·m^2^·kg^−1^, respectively. Considering that the saturation magnetization of bulk maghemite is 76 A·m^2^·kg^−1^ [43,44], the MNPs exhibit a rather low saturation magnetization value. This is due to the coating of nonmagnetic organic ligands, which may reduce the total magnetization value of MNPs and the magnetically disordered layer on the surface of the nanoparticles [45,46].

### 3.5. Colorimetric Detection of H_2_O_2_ Based on Magnetic Hydrogels

**Figure 3:**
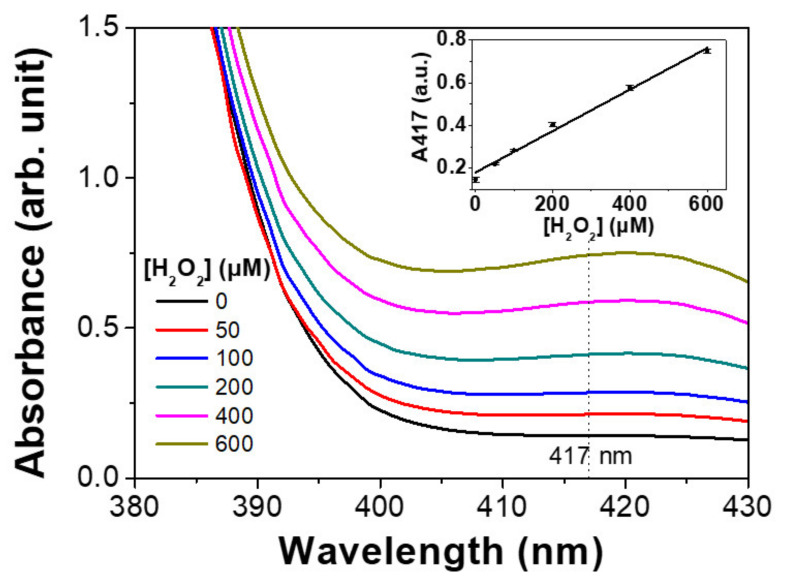
Colorimetric detection of H_2_O_2_ using magnetic hydrogels. Absorption spectra of 2,2–azino–bis (3–ethylbenzothiazoline–6–sulfonic acid) diammonium salt (ABTS) oxidation in magnetic hydrogels as a function of H_2_O_2_ concentration. Inset: absorbance at 417 nm as a function of H_2_O_2_ concentration.

Magnetic hydrogels containing MNPs exhibit peroxidase-like activity, which enables their potential use in H_2_O_2_-mediated bioassays. Colorimetric detection of H_2_O_2_ was performed to demonstrate the catalytic activity of the magnetic hydrogels. ABTS was used as a substrate in the assay, because both ABTS and its oxidized form are highly water-soluble and move freely inside and outside hydrogels. The magnetic hydrogels catalyzed the oxidation of ABTS by H_2_O_2_, and the absorbance of oxidized ABTS was linearly correlated with H_2_O_2_ concentration (Figure 3).

Water-dispersible MNPs with dimensions on the order of 10 nm are superparamagnetic, but they cannot be separated completely from aqueous media by applying moderate magnetic fields. On the contrary, nanoparticles that are above 30 nm in size are ferromagnetic and magnetically separable, but they cannot be re-dispersed after magnetic separation owing to their remnant magnetization [47]. To enhance separation efficiency while retaining the superparamagnetic character, the fabrication of magnetic nanoparticle clusters was proposed [48], and the encapsulation of MNPs inside hydrogels resulted in clusters of superparamagnetic nanoparticles.

Magnetically separable hydrogels offer several important advantages in H_2_O_2_-mediated assays. One advantage is that these particles can be easily separated from the test sample with a magnet to stop a given reaction step. This provides sufficient time for measurement procedures while minimizing signal bias after quenching the reaction. Another advantage is that complete removal of these particles reduces signal noise due to the absence of MNPs in the remaining solution. The background absorbance of free MNPs at the assay wavelength in the visible region can be non-negligible and variable (Figure S6). One drawback is that the overall reaction rate using magnetic hydrogels was lower than that using free MNPs. Since the catalytic reaction occurs on the nanoparticles trapped inside the hydrogels, the oxidized ABTS must move through the hydrogel matrix before an accurate absorbance measurement can be taken.

**Figure 4:**
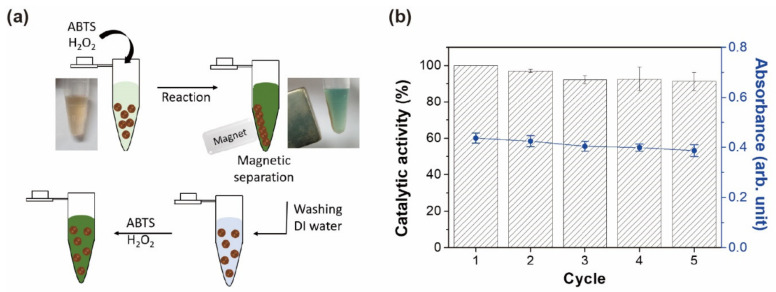
Catalytic activity of reusable magnetic hydrogels. (**a**) Initial use and subsequent reuse of magnetic hydrogels for detecting H_2_O_2_-mediated reactions. (**b**) Catalytic activity of magnetic hydrogels after multiple cycles of assay reaction and magnetic separation. Absorbance at 417 nm was used to calculate the catalytic activity.

One interesting observation is that magnetic hydrogels demonstrated the ability to be reused multiple times after simple collection and washing with DI water (Figure 4a). This reusability test was performed on a larger scale using a high dose of magnetic hydrogels (3-fold) and a prolonged magnetic field than the assays on microplates, allowing efficient removal of the solution generated in each cycle. Over five repeats of the same assay steps, the catalytic activity for ABTS and H_2_O_2_ maintained over 90% of the initial activity (Figure 4b). The sustained activity is a product of both the stability of nanoparticles against H_2_O_2_ and the structural advantage of hydrophilic hydrogels. Since freely dispersed nanoparticles can aggregate or become deactivated in catalytic reactions, it is difficult to maintain activity over the course of one or multiple assay steps. By synthesizing a PEG-based hydrogel containing uniformly distributed (yet securely isolated) MNPs, such failure modes were not observed to a significant extent.

Drying is a conventional method to store hydrogels because removing water can reduce the overall volume and minimize biological contamination and degradation. Magnetic hydrogels were dried by freeze-drying, and then they were rehydrated with DI water for the investigation on the catalytic performance. The rehydrated magnetic hydrogels showed 90% catalytic activity in the colorimetric detection of H_2_O_2_ compared to the undried ones (Figure S7). These results showed that magnetic hydrogels can be stored in dry form and used in assays after simple rehydration.

### 3.6. Colorimetric Detection of Glucose Based on Magnetic Hydrogels

**Figure 5:**
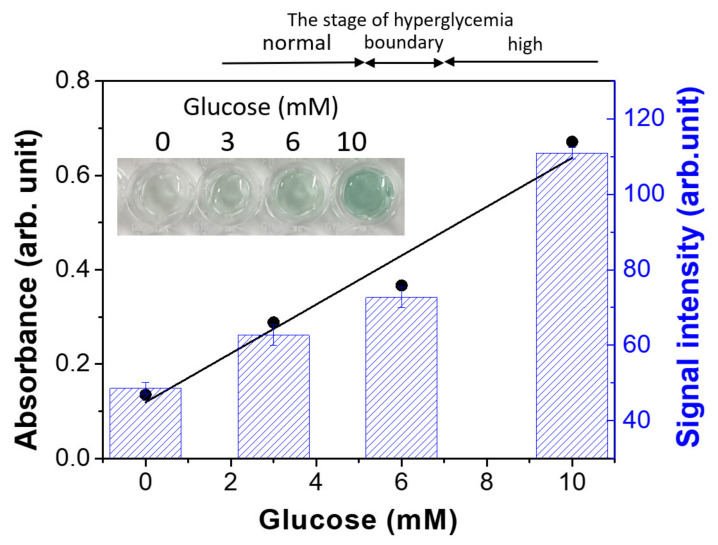
Colorimetric detection of glucose concentration using magnetic hydrogels. Absorbance at 417 nm (black vertical axis) and signal intensity (blue vertical axis) are captured with a smartphone and analyzed in ImageJ. Inset: colorimetric signal generated by different glucose concentrations.

Glucose detection is a popular H_2_O_2_-mediated bioassay [49], yet there is still great demand for simple and facile methods for diabetes monitoring. Colorimetric assays have received much attention because of their cost-effective and user-friendly outputs, such as sample detection that can be easily captured and interpreted using smartphones [50]. For glucose detection, GOx was used to catalyze the oxidation of glucose. H_2_O_2_ was generated by the reduction of oxygen, depending on the concentration of glucose. Glucose model solutions were prepared with representative levels of glucose corresponding to the normal, boundary, and high stages of hyperglycemia (normal: ≤5.6 mM, boundary: 5.6–7 mM, and high: >7 mM) [51]. The absorbance from the resulting solutions was measured at 417 nm (Figure S8), and a photographic image was taken by a smartphone (Figure 5). The absorbance showed a linear correlation with glucose concentration in the solution, and the “hyperglycemia” model solution exhibited a green color that was distinguishable by the naked eye. The intensity of each well from the photograph was calculated using ImageJ, with the hyperglycemia signal being distinguishable from the normal and boundary signals.

## 4. Conclusions

In this study, we have demonstrated that a photoinitiator’s absorbance property can drive the synthesis of uniform and fully polymerized magnetic hydrogels. The effects of absorbance on the morphology and structural uniformity of the synthesized magnetic hydrogels were investigated by comparing two photoinitiators with highly dissimilar absorbance spectra. We found that only the photoinitiator with high absorbance (in the region of interest) achieves synthesis of magnetic hydrogels with uniform internal structure and without shape deformation. VSM measurements were used to confirm that both MNPs and magnetic hydrogels have superparamagnetic properties. The magnetic hydrogels were used in assays for colorimetric H_2_O_2_ detection with sustainable (and repeatable) catalytic activity. The magnetic hydrogels were also used to achieve colorimetric glucose detection, with distinguishable results based on specific health state.

Considering the simplicity of synthesizing uniform magnetic hydrogels via photopolymerization, we anticipate our method to be widely used in the synthesis of other inorganic material-embedded polymers without risk of aggregation and/or sedimentation. Furthermore, we believe that the synthesized magnetic hydrogels will provide a versatile platform for H_2_O_2_-mediated colorimetric assays, with easy sample isolation and highly sustainable catalytic activity.

## Supplementary Materials

The following are available online at https://www.mdpi.com/1996-1944/13/19/4401/s1, Figure S1: (**a**) A schematic diagram for the synthesis of water-dispersible magnetite nanoparticles (MNPs). (**b**) TEM image (left) and the plot of the volume versus hydrodynamic size distribution (right) of MNPs. The average diameters measured by the TEM image and average hydrodynamic diameter measured by DLS are 12 nm and 16 nm, respectively, Figure S2: Particle histogram showing the circularity of magnetic hydrogels synthesized with a photoinitiator, either a Darocur 1173 or LAP. Ten particles were measured for each photoinitiator via the ImageJ software. A circularity of 1.0 represents a perfect spherical morphology, Figure S3: Magnetic hydrogels embedding 5 mg/mL of MNP concentration synthesized with LAP and a circularity histogram of the microparticles, Figure S4: Dot plots of fluorescence intensity of individual magnetic hydrogels synthesized with either Darocur 1173 or LAP. Twenty microparticles for each photoinitiator were measured via the ImageJ software, Figure S5: VSM measurement results. M–H curves of freeze-dried MNPs (left) and magnetic hydrogels (right). The concentration of magnetic hydrogels before drying was 4 mg Fe/mL. M versus 1/B and a linearly fitted line at a magnetic field from 0.2 to 0.5 T are presented in each graph, Figure S6: Comparative absorbance spectra of free MNPs (red) and magnetic hydrogels (black) mixed with ABTS. Each solution contains 160 μL of sodium acetate buffer (0.2 M, pH 4.0), 20 μL of ABTS (60 mM), and 20 μL of free MNPs or magnetic hydrogels (0.2 Fe mg/mL), and the external magnetic field was applied for 10 s before the measurement, Figure S7: Comparative catalytic activity of freeze-dried magnetic hydrogels after rehydration with DI water. Hydrogels (20 μL, 0.2 Fe mg/mL) was incubated with ABTS (20 μL, 60 mM) and H_2_O_2_ (20 μL, 5 mM) in sodium acetate buffer (0.2 M, pH of 4.0). Absorbance at 417 nm was used to calculate the catalytic activity, Figure S8: Absorbance spectra for dose-responsive glucose detection by magnetic hydrogels with GOx.
