# Supplementary material for: Photopolymerization-Based Synthesis of Uniform Magnetic Hydrogels and Colorimetric Glucose Detection
Source: Materials (Basel). 2020 Oct 2;13(19):4401. doi: 10.3390/ma13194401 (PMC7579115; doi:10.3390/ma13194401)
Supplement: Supplementary file 1 [file materials-13-04401-s001.pdf]

materials

MDPI

Supplementary Materials

Photopolymerization-Based Synthesis of Uniform Magnetic Hydrogels and Colorimetric Glucose Detection

Seok Joon Mun [1,†], Donghyun Ko [2,†], Hyeon Ung Kim [1], Yujin Han [2], Yoon Ho Roh [1], Bong-Geun Kim [2], Hyon Bin Na [2,*] and Ki Wan Bong [1,*]

[1] Department of Chemical and Biological Engineering, Korea University, 145 Anam-ro, Seongbuk-gu, Seoul 02841, Korea; msj95@naver.com (S.J.M.); rlagus2009@gmail.com (H.U.K.); yoonho90@korea.ac.kr (Y.H.R.)
[2] Department of Chemical Engineering, Myongji University, Yongin, Gyeonggi-do 17058, Korea; rhehd1992@naver.com (D.K.); padug2@naver.com (Y.H.); persson@naver.com (B.-G.K.)
* Correspondence:hyonbin@mju.ac.kr (H.B.N.); bong98@korea.ac.kr (K.W.B.)
† The authors are equal contribution to this work.

Received: 4 September 2020; Accepted: 30 September 2020; Published: date

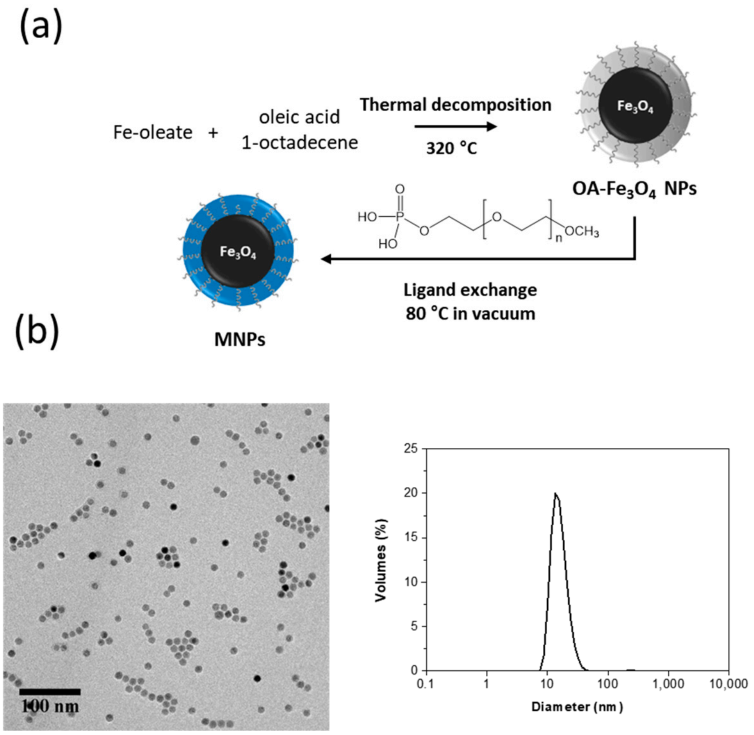

Figure S1. (**a**) A schematic diagram for the synthesis of water-dispersible magnetite nanoparticles (MNPs). (**b**) TEM image (left) and the plot of the volume versus hydrodynamic size distribution (right) of MNPs. The average diameters measured by the TEM image and average hydrodynamic diameter measured by DLS are 12 nm and 16 nm, respectively.

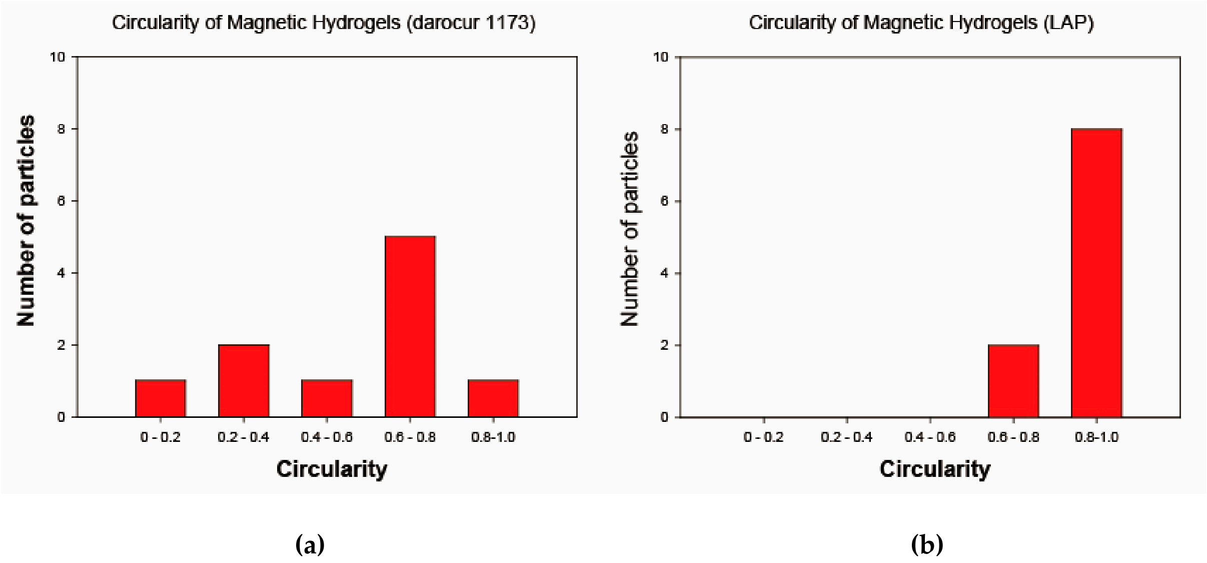

Figure S2. (**a**) Particle histogram showing the circularity of magnetic hydrogels synthesized with Darocur 1173. (**b**) Particle histogram showing the circularity of magnetic hydrogels synthesized with LAP. Ten particles were measured for each photoinitiator via ImageJ software. A circularity of 1.0 represents a perfect spherical morphology.

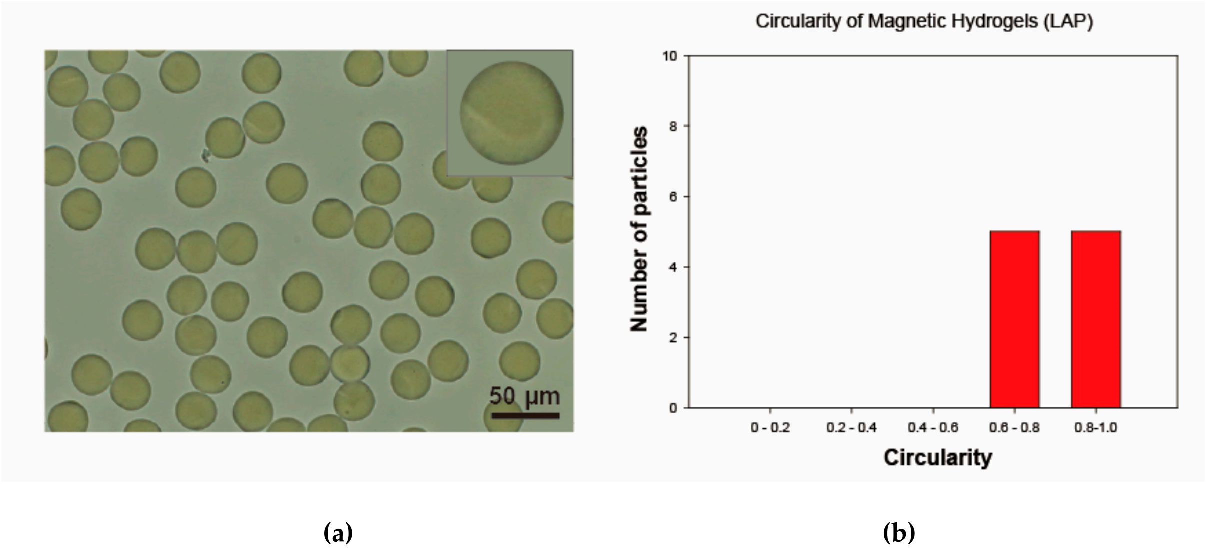

Figure S3. (**a**) Magnetic hydrogels embedding 5 mg/mL of MNP concentration synthesized with LAP.(**b**) A circularity histogram of the synthesized magnetic hydrogels.

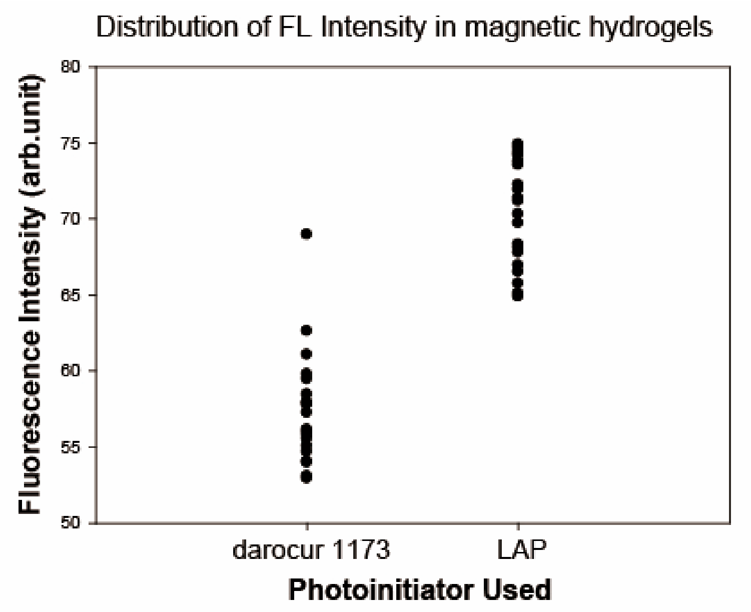

Figure S4. Dot plots of fluorescence intensity of individual magnetic hydrogels synthesized with either Darocur 1173 or LAP. 20 microparticles for each photoinitiator were measured via ImageJ software.

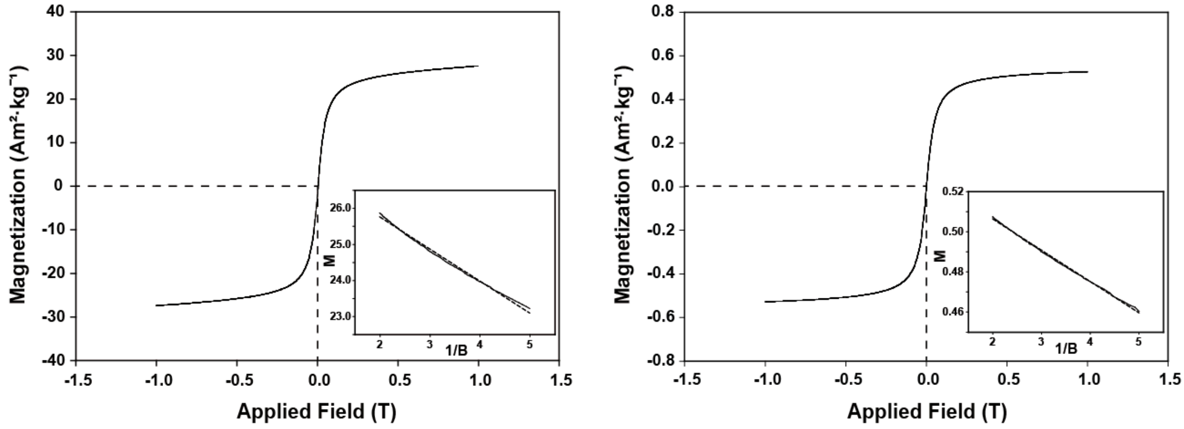

(a) (b)

Figure S5. VSM measurement results. M–H curves of freeze-dried (**a**) PEG-coated MNPs and (**b**) magnetic hydrogels. The concentration of magnetic hydrogels before drying was 4 mg Fe/mL. M versus 1/B and a linearly fitted line at a magnetic field from 0.2 T to 0.5 T are presented in both graphs.

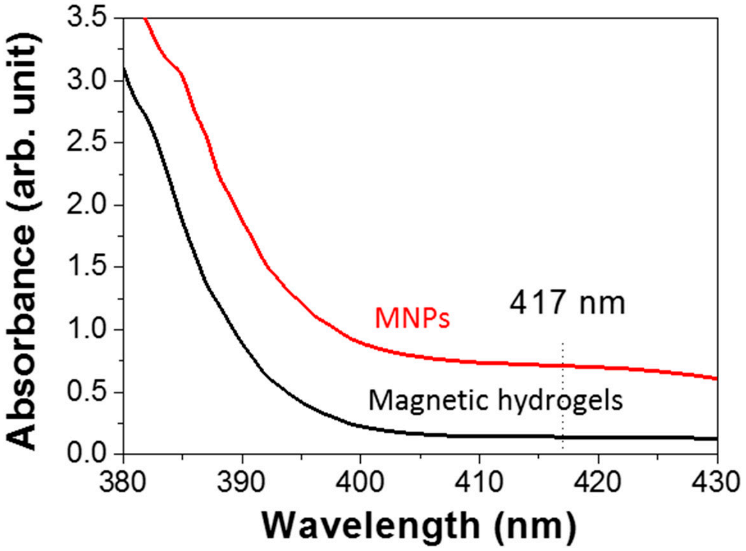

Figure S6. Comparative absorbance spectra of free MNPs (red) and magnetic hydrogels (black) mixed with ABTS. Each solution contains 160 μL of sodium acetate buffer (0.2 M, pH 4.0), 20 μL of ABTS (60 mM), and 20 μL of free MNPs or magnetic hydrogels (0.2 Fe mg/mL), and the external magnetic field was applied for 10 s before the measurement.

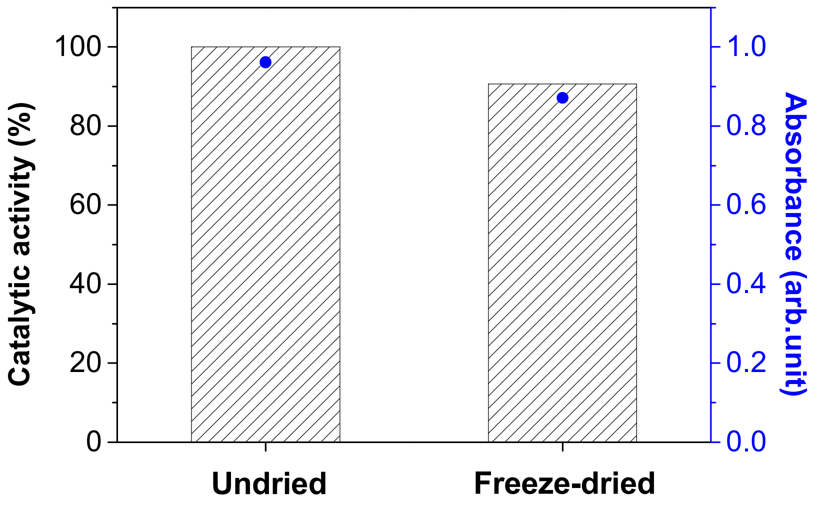

Figure S7. Comparative catalytic activity of freeze-dried magnetic hydrogels after rehydration with DI water. Hydrogels (20 μL, 0.2 Fe mg/mL) was incubated with ABTS (20 μL, 60 mM) and H_2O_2 (20 μL, 5 mM) in sodium acetate buffer (0.2 M, pH 4.0). Absorbance at 417 nm was used to calculate the catalytic activity.

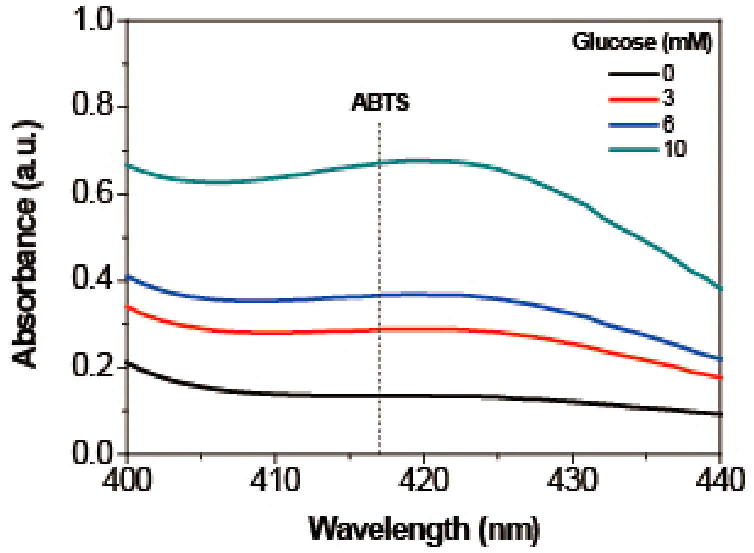

Figure S8. Absorbance spectra for dose-responsive glucose detection by magnetic hydrogels with GOx.
